# Auditory Short-Term Memory Behaves Like Visual Short-Term Memory

**DOI:** 10.1371/journal.pbio.0050056

**Published:** 2007-02-20

**Authors:** Kristina M Visscher, Elina Kaplan, Michael J Kahana, Robert Sekuler

**Affiliations:** 1 Volen Center for Complex Systems, Brandeis University, Waltham, Massachusetts, United States of America; 2 Department of Psychology, University of Pennsylvania, Philadelphia, Pennsylvania, United States of America; University of Maryland, United States of America

## Abstract

Are the information processing steps that support short-term sensory memory common to all the senses? Systematic, psychophysical comparison requires identical experimental paradigms and comparable stimuli, which can be challenging to obtain across modalities. Participants performed a recognition memory task with auditory and visual stimuli that were comparable in complexity and in their neural representations at early stages of cortical processing. The visual stimuli were static and moving Gaussian-windowed, oriented, sinusoidal gratings (Gabor patches); the auditory stimuli were broadband sounds whose frequency content varied sinusoidally over time (moving ripples). Parallel effects on recognition memory were seen for number of items to be remembered, retention interval, and serial position. Further, regardless of modality, predicting an item's recognizability requires taking account of (1) the probe's similarity to the remembered list items (summed similarity), and (2) the similarity between the items in memory (inter-item homogeneity). A model incorporating both these factors gives a good fit to recognition memory data for auditory as well as visual stimuli. In addition, we present the first demonstration of the orthogonality of summed similarity and inter-item homogeneity effects. These data imply that auditory and visual representations undergo very similar transformations while they are encoded and retrieved from memory.

## Author Summary

Memories are not exact representations of the past. But can we say that all our senses are equally reliable (or unreliable) sources for memory? We performed a series of experiments to test that proposition. Sound and light are processed by different receptors and neural pathways in the brain. Previous comparisons of auditory and visual memory have done little to place on equal footing the stimuli that will be remembered, limiting the ability to truly compare the two processes. However, using current knowledge of how these sensations are represented in the nervous system, we created auditory and visual stimuli of similar complexity and that undergo similar initial processing by the nervous system. We then used these well-matched stimuli to examine memory for studied lists of either auditory or visual items. Using behavioral measures and a computational model for list memory, we show that memory representations are altered similarly for both hearing and vision. We found that auditory and visual memory exhibit striking parallels in terms of how memory is affected by all the parameters we changed in this experiment. These results imply that auditory and visual short-term memory employ similar mechanisms.

## Abstract 3

The mechanisms by which memories are encoded and retrieved share common principles between the visual and auditory systems of humans.

## Introduction

In the past decade, cognitive science has spawned some powerful computational models for both the large-scale and detailed structure of many fundamental phenomena, including categorization and recognition. These models have enjoyed considerable success, particularly in accounting for recognition of simple visual stimuli, such as sinusoidal gratings and chromatic patches [1–3], and more complex visual stimuli, such as realistic synthetic human faces [4]. By exploiting stimuli whose properties can be easily manipulated, but resist consistent verbal rehearsal strategies [5], researchers can formulate and test detailed predictions about visual recognition memory.

To date, this effort has focused on vision, raising the possibility that the properties of recognition memory revealed thus far might be modality specific and therefore of limited generality. There are several prerequisites that must be satisfied before another sensory modality can be addressed in a comparable fashion. First, a suitable task must be found; second, a family of stimuli must be identified that can be parametrically varied along dimensions thought to be encoded in memory. In addition, baseline memory performance must be comparable across modalities, and the effect of early perceptual processing on the stimulus representations must be similar. Failure to satisfy any of these prerequisites would undermine inter-modal comparisons of memory.

We decided to use Sternberg's recognition memory task, which had been used previously with visual stimuli and whose properties were well understood [6]. We then identified a family of auditory stimuli—moving ripple sounds—whose attributes resembled ones that had proven useful in modeling visual recognition memory. These auditory stimuli vary sinusoidally in both time and in frequency content, and are generated by superimposing sets of tones whose intensities are sinusoidally modulated. Until now, these stimuli have been mainly used to characterize the spectro-temporal response fields of neurons in mammalian primary auditory cortex [7–9], but because their spectro-temporal properties resemble those of human speech [7,10], moving ripple stimuli are well suited to probe human speech perception and memory with minimal contamination by semantic properties or by the strong boundaries between existing perceptual categories [11].

This selection of stimuli was influenced by previous attempts to compare auditory and visual memory. Some of those attempts used auditory and visual stimuli that differed substantially in their early sensory processing, but shared semantic representations [12]. For example, Conrad and Hull's classic study compared memory for a list of digits presented either visually or as spoken items [13]. Initial processing differs tremendously for the two types of inputs, indicating that differences in memory may be due to the divergent initial processing. Further, with stimuli like these, once the items have been encoded into verbal form for storage in memory, shared semantic processes may obscure any fundamental differences in memory for the two modalities. Other experiments use stimuli that arguably are free from semantic influences, yet still fail to equate the early stages of processing required by the stimuli [14].

We examined short-term memory for auditory and visual stimuli whose early sensory processing is comparable. Finding comparable stimuli across modalities is difficult, as it may initially seem incontrovertible that the brain operates differently upon auditory and visual inputs. Certainly the initial stages of processing by the modalities' respective receptors differ from one another in many ways. However, the transformations performed by the nervous system on the information generated by the auditory and visual receptors appear to be very similar [7,15]. Starting from each modality's sensory receptors and continuing to the modality's respective processing networks within the cerebral cortex, analogs between hearing and vision have been noted by several researchers [7,9,16,17]. To take a few examples, adjacent sensory receptors in the cochlea of the ear detect neighboring frequencies of sound the same way adjacent sensory receptors in the retina of the eye respond to light from neighboring locations in space. This analogy extends to the retinotopic/tonotopic structure and receptive fields of auditory and visual cortex.

**Figure 1:**
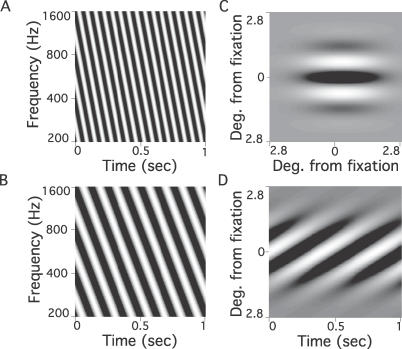
Auditory, Stationary Visual, and Moving Visual Stimuli (A and B) Spectro-temporal plots of two auditory moving ripple stimuli. The horizontal axis shows time in seconds; the vertical axis shows frequency content in Hertz (Hz). Brightness indicates the amplitude at a particular frequency and time; white represents sounds of greater amplitude. Modulations in frequency content are referred to as spectral density, defined by sinusoidal frequency Ω; modulations over time are referred to as the ripple's velocity, defined by sinusoidal frequency *w*. The ripple velocity for (A) is 16 Hz, and for (B), it is 8 Hz. (C) Image representing the static grating stimuli used. Horizontal and vertical axes represent actual spatial extent, in degrees visual angle from fixation, of visual stimuli. (D) Space–time plot of one moving grating stimulus. The horizontal axis shows time in seconds; the vertical axis shows the horizontal dimension in space *(y)*. Brightness indicates the luminance at that point on the display. Moving visual gratings looked like (C) in which the bars drifted upwards over time.

Both moving ripples and Gabor patches vary sinusoidally along the dimensions that primary sensory neurons encode. These stimuli are described in Figure 1. Moving further along the processing hierarchy, it appears that primary auditory cortex responds to moving ripple stimuli analogously to the way primary visual cortex responds to Gabor patches: a few neurons respond robustly to the stimulus, but most are relatively quiet [9]. The sets of stimuli, therefore, are very well matched in terms of early sensory processing. In addition, to decrease reliance upon verbal rehearsal, these unfamiliar stimuli can be varied continuously, and do not support readily available verbal or semantic labels [5]. So we should expect results to be minimally influenced by semantic relationships among stimuli.

Finally, to promote comparability in the difficulty of the memory task with auditory or visual stimuli, we adopted a strategy introduced by Zhou and colleagues [18]. Recognizing that the similarity relationships among visual stimuli strongly influenced recognition memory, those researchers adjusted each participants' memory test stimuli according to that participant's discrimination threshold. Their aim was to minimize individual differences on the memory task. We took the procedure one step further, adjusting stimuli separately within each modality according to each participant's discrimination threshold for that modality. This was meant to equate for both auditory and visual modalities the powerful influence that similarity exerts on memory.

We present the results of two experiments. Experiment 1 assessed several basic properties of recognition memory for ripple stimuli and memory for Gabor patches; Experiment 2 used ripple stimuli to isolate the effects of summed probe-item similarity and inter-item homogeneity. The design of Experiment 2 was meant to orthogonalize these two potential influences on recognition memory, allowing the effects of summed similarity and inter-item homogeneity to be explored independently. A previously proposed model for visual memory, the Noisy Exemplar Model (NEMo) was fit to the data [1]. Because so many trials were required for each case, and because the NEMo has been shown previously to fit data for visual stimuli quite well [1], only auditory stimuli were used in Experiment 2.

## Results

### Experiment 1: Basic Properties of Short-Term Recognition Memory

Experiment 1 measured short-term recognition memory for moving ripple stimuli and for both moving as well as stationary Gabor patches. We used a variant of Sternberg's recognition task [6,19]. On each trial, one to four stimuli were sequentially presented, followed after some retention interval by a probe. The participants' task was to identify whether the probe matched any of the items presented in the list, pressing a button to indicate their choice. The use of the Sternberg paradigm for auditory stimuli allows comparisons to the many studies that have used the same paradigm with visual stimuli [1,19,20].

Both moving and static visual Gabor patches were tested because although moving Gabor patches change in time similarly to the ripple sounds, their stationary counterparts have been extensively studied in psychophysical examinations of memory [1]. We examined several basic properties of short-term memory for auditory and visual stimuli: the effect of the number of stimuli that must be remembered (list length), the interval over which those stimuli must be remembered (retention interval), and the serial position of the stimulus matching a probe.

Each participant's data from trials of a given list length and retention interval were averaged to obtain a proportion correct for that combination of conditions. These were compared across participants using standard parametric statistics. Proportion correct measures were used rather than, for example, *d′* measures because in this case, the assumption that variances associated with target (probe matches a list item) and lure (probe does not match a list item) trials are identical is probably not defensible, as the range of summed probe-item similarities for target trials is much smaller than for lures (as by definition, Target trials always include a stimulus that is identical to the probe, with a similarity equal to 1) [21].

#### Effects of length of the study list, retention interval, and serial position.

**Figure 2:**
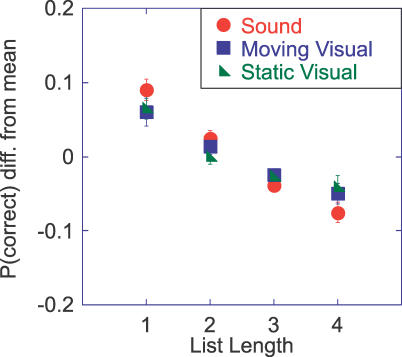
Effect of Length of the Study List Difference from mean proportion correct responses (*P*(correct) diff) is plotted as a function of length of the study list. Statistics show a significant effect of stimulus type and list length, but no interaction between them. The mean proportions correct subtracted for each stimulus type were: sound, 0.72; moving visual, 0.61; and stationary visual, 0.59.

Figure 2 shows the proportion of correct responses made as a function of the length of the study list. Error bars show the within-participant standard error of the mean, taking out between-participant variability and between–stimulus type variability, and indicate results of the effect of list length by analysis of variance (ANOVA) [22,23]. Note that as the number of elements in the list increases, participants are correct less often. The effect of list length is significant in a 3 × 4 (stimulus types by list lengths) ANOVA (*F*_3,39_ = 32.5, *p* < 0.0001). In addition, the overall proportion correct is different depending on the stimulus type (*F*_2,26_ = 29.2, *p* < 0.0001). Participants' proportion correct was overall larger for the ripple sounds than for the grating stimuli in all conditions. This difference indicates that the sound stimuli chosen were more easily discriminable than the visual stimuli. The interaction of the effect of list length with stimulus type was nonsignificant (*F*_6,78_ = 1.53, *p* = 0.18,).

**Figure 3:**
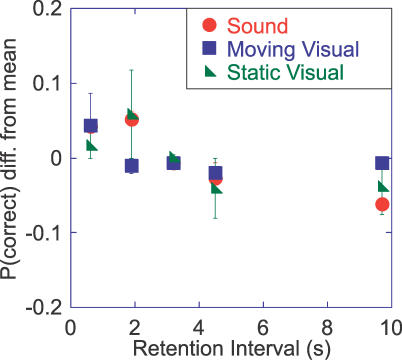
Effect of Retention Interval Difference from mean proportion correct responses (*P*(correct) diff) is plotted as a function of the retention interval. Proportion correct decreases modestly with retention intervals up to 9.7 s. The mean proportions correct subtracted for each stimulus type were: sound, 0.77; moving visual, 0.66; and stationary visual, 0.62.

Figure 3 shows that even as the retention interval goes to 9.7 s, the proportion correct changes less than 10%. This change is nonetheless significant in a 3 × 5 (stimulus types by retention intervals) ANOVA (*F*_4,52_ = 10.76, *p* < 0.0001). As with all conditions, proportion correct was overall larger for ripple sounds (*F*_4,52_ = 26.12, *p* < 0.0001). The interaction between stimulus type and retention interval was only marginally significant (*F*_8,104_ = 2.02, *p* = 0.051). Error bars show the within-participant standard error of the mean, taking out between-participant variability and between–stimulus type variability, indicating results of the effect of retention interval by ANOVA [22,23].

**Figure 4:**
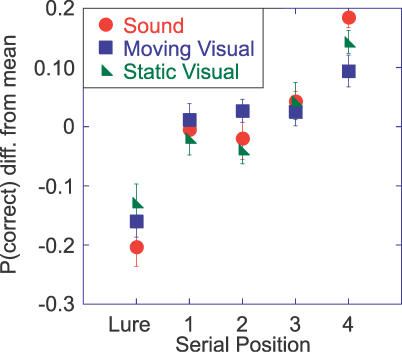
Effect of Serial Position Difference from mean proportion correct responses (*P*(correct) diff) is plotted as a function of the serial position of the matching study item. Data are shown for the case in which four stimuli were presented. Statistics show a significant effect of serial position and list length, but no interaction between them. The mean proportions correct subtracted for each stimulus type were: sound, 0.72; moving visual, 0.62; and stationary visual, 0.61.

Figure 4 shows the effect of serial position on recognition rate. For clarity, only the data for the four-stimulus case are shown. Effects were similar for the other list lengths. The most recently presented stimulus is recognized more often when it matches the probe than are earlier stimuli. For lists of four items, a 3 × 4 (stimulus types by serial positions) ANOVA showed no significant interaction between stimulus type and serial position (*F*_6,78_ = 1.3, *p* = 0.26). However, there was a highly significant effect of serial position (*F*_3,39_ = 24.3, *p* < 0.0001), and an effect of stimulus type (*F*_2,26_ = 10.1, *p* <0.001). Error bars show the within-participant standard error of the mean, taking out between-participant variability and between–stimulus type variability, indicating results of the effect of serial position by ANOVA [22,23].

There appears to be a slight trend towards a greater recency effect in the case of auditory stimuli than for the visual stimuli, so that later serial positions are remembered more accurately. Although this does not reach significance in the list length 4 or 2 cases, it is marginally significant in the list length 3 case, in which a 3 × 3 (stimulus types by serial positions) ANOVA reveals a slight interaction between stimulus type and serial position (*F*_4,52_ = 2.7, *p* = 0.04).

### Experiment 2: Effect of Inter-Item Homogeneity and Summed Similarity on Memory for Ripple Sounds

Previous studies have shown that short-term recognition memory for visual stimuli can be understood using the NEMo introduced by Kahana and Sekuler [1]. Experiment 2 directly tested this model's predictions for memory for moving ripple sounds, and compared these results to previous results obtained with visual stimuli. This experiment was crafted so that the key assumptions of the model, effects of inter-item homogeneity and summed similarity, could be explored in a model-free way, while also allowing data to be fit to NEMo for a more quantitative assessment of these effects. The next section explains the logic of the experimental design.

#### NEMo.

Contemporary, exemplar-based memory models, such as the Generalized Context Model [24], assume that when participants judge whether a probe stimulus replicated one of the preceding study items, their judgments reflect the summed similarity of the probe to each study item in turn, (summed probe-item similarity), rather than the similarity of the probe to its one most-similar study item [1,25]. In addition, recent studies have shown that the similarity between the individual items to be remembered, the inter-item homogeneity, also has an effect on participants' performance. When items in memory are more homogeneous, participants make relatively few false alarms; when items in memory are less homogeneous, rate of false alarms increases [1–4]. This could indicate that the participant adopts a less-strict criterion on trials in which the items are less homogeneous, or it may indicate that the memory representation is less fine-grained on trials in which stimuli are more different from one another. This effect has been found with a range of various visual stimuli, including oriented, compound sinusoidal gratings [1,3], realistic, synthetic human faces [4], and color samples [2]. Because all the memory stimuli assayed thus far were visual, it may be that the effect of homogeneity on memory is modality specific, a possibility that we examined in the current studies.

If the inter-item homogeneity effect held for auditory stimuli, this would support the idea that the mechanism supporting the effect of homogeneity is shared by both auditory and visual memory. Ripple stimuli are useful for examining the effects of homogeneity and summed probe-item similarity due to their many parallels with Gabor patches, and their parametric variability.

#### Summed probe-item similarity.

**Figure 5:**
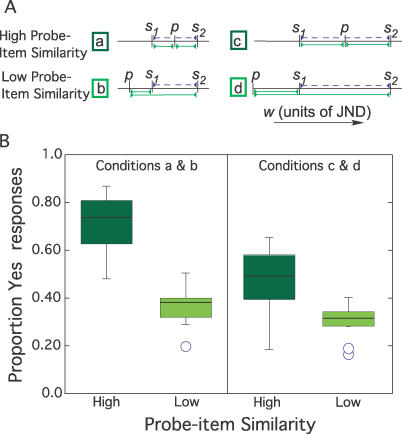
Effect of Probe-Item Similarity (A) A schematic diagram of four stimulus conditions. These examine effects of summed probe-item similarity on participants' report of having seen a stimulus. Conditions a–d keep inter-item homogeneity constant (pairs a & b and c & d) while changing summed probe-item similarity. These diagrams show the relationships between conditions, rather than the actual values stimuli may take. Throughout, summed probe-item similarity is denoted in green, whereas inter-item similarity is denoted in blue. Conditions in the first row have high summed similarity (indicated by the shorter green solid bars). These are identical to their pairs (b and d, respectively) in the second row in terms of inter-item homogeneity (indicated by the length of the blue dashed bar), and similarity of the probe to the closest item (shorter of the two green solid bars). The second row shows cases of lower summed similarity (longer green solid bars). (B) The results of the experiment. For each pair of otherwise matched stimuli, when summed similarity is larger, participants are more likely to indicate that a probe has been seen before (*p* < 0.01). These box plots show the median (thick bar), and boxes include the middle 50% of data. The whiskers include all data points that are not outliers. Outliers are shown as circles, and defined as those points more than 1.5 times the interquartile range from the median. Light and dark green indicate high (conditions a & c) and low (conditions b & d) summed similarity, respectively.

Several models of visual short-term memory (including NEMo) posit that participants use information about the summed similarity between the probe and all the remembered items, rather than just the item most similar to the probe, to make a judgment about whether the probe was seen before [1,26]. Two pairs of conditions were created (shown in Figure 5A) that were similar in all respects, but the summed probe-item similarity varied between the two conditions in the pair.

Figure 5B shows that greater summed probe-item similarity (left side of each pair) predicts greater probability of a *Yes* response (paired T-test for conditions a and b showed *p* < 0.00001, for c and d, *p* < 0.01). This experiment controlled for the similarity between the probe and the stimulus closest to it, as well as the inter-item homogeneity of the list, therefore indicating that the observed effects are due to summed probe-item similarity rather than other variables.

#### Inter-item homogeneity.

As noted in the Introduction, one goal in performing this experiment was to determine whether and how the homogeneity between items in memory influences participants' subsequent recognition for sounds. With this in mind, stimulus conditions were created that varied inter-item homogeneity while other factors (summed similarity between the probe and each item, and similarity between the probe and the item most similar to it) were held constant. This allowed the effect of inter-item homogeneity to be explored independently.

**Figure 6:**
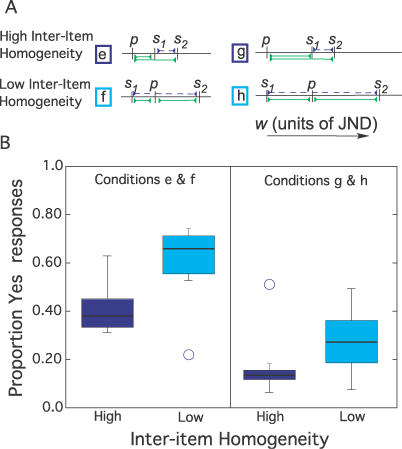
Effect of Inter-Item Similarity (A) A schematic diagram of four stimulus conditions. These examine effects of inter-item homogeneity on participants' report of having seen a stimulus. Conditions e & f keep summed probe-item similarity constant (indicated by the total length of green bars for each condition) while changing inter-item homogeneity (length of blue dashed bar). Conditions g & h do the same, but for a different summed probe-item similarity. These diagrams show the relationships between conditions, rather than the actual values stimuli may take. As in all figures, inter-item similarity is denoted in shades of blue, whereas summed probe-item similarity is in green. (B) The results of the experiment. For each pair of otherwise matched stimuli, when stimulus items are more homogeneous (dark blue), participants are less likely to indicate that a probe has been seen before than if the stimulus items are less homogeneous (light blue) (*p* < 0.01). Box and whisker plot conventions are as described in the caption for Figure 5B.

Figure 6 shows that as inter-item homogeneity is increased, a probe is less likely to attract a *Yes* response (paired T-test for conditions e and f showed *p* < 0.00001, for c and d, *p* < 0.01). Note that the experiment controlled for similarity between the probe and the list items, as well as the similarity between the probe and the item closest to it.

#### Perceptual similarity.

**Figure 7:**
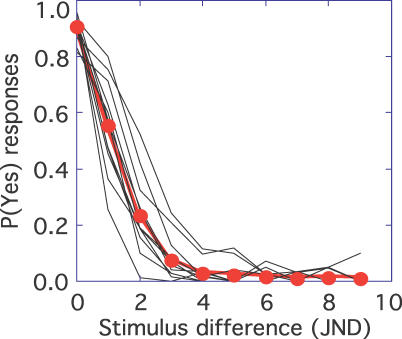
Proportion *Yes* Responses as a Function of Difference between Probe and To-Be-Remembered Item (in Units of JND) Twelve participants' data are shown in thin lines, with the average in thick red. These curves are well described by the exponential function of Equation 4. This function relates actual stimulus values to their mental representations. *P*(yes) responses indicate proportion of *Yes* responses.

The perceived similarity between ripple sounds is monotonic with their physical difference. Figure 7 shows data from the cases in which a single list item was presented and followed immediately by a probe. When the probe matched the stimulus, participants were very likely to respond “*Yes,* there was a match.” As the difference between the stimuli increased, the proportion of *Yes* responses decreased monotonically, indicating a reduced likelihood that the probe would be confused with the stimulus that preceded it.

### Computational Models: Context Affects Memory for Sounds

Figures 5 and 6 show that inter-item homogeneity and summed probe-item similarity both affect memory for complex sounds. This result is analogous to that observed for visual stimuli [1,2,4,18]. By fitting the same computational models to these auditory data and visual memory data, we can more sensitively examine whether the cognitive processing undergone by auditory and visual representations are similar.

The data for Experiment 2 were fit to the models described in the Methods section: a three-parameter model that does not take into account inter-item homogeneity, a four-parameter model that adopts values describing perceptual similarity based on participants' performance when list length is 1 (Figure 7), and a five-parameter model including inter-item homogeneity effects, and not assuming that perceptual similarity can be based on participants performance for list length 1.

Table 1 shows the parameter values produced by model fits to the combined data for 12 participants. Fits were made for individual participants as well, and the parameters are similar in the individual participant fits and the fits to the average. The *τ* and *σ* parameters showed most variability across participants. Models in which the τ parameter was estimated from an independent dataset in which study lists comprised just one item are indicated. The value for *A* calculated from data with list length 1 was 0.93 for the data averaged over participants, with individual participant values ranging from 0.88 to 1.02. The value for *τ* calculated from data with list length 1 ranged from 0.43 to 1.41 across participants. When *τ* was allowed to vary in the five-parameter model, the value ranged from 0.97 to 3 (the maximum of the allowed range) across participants. The parameter *τ* and the criterion *C* have somewhat of a reciprocal relationship mathematically, and so their values depend on one another: as *C* decreases, *τ* increases.

**Table 1:**
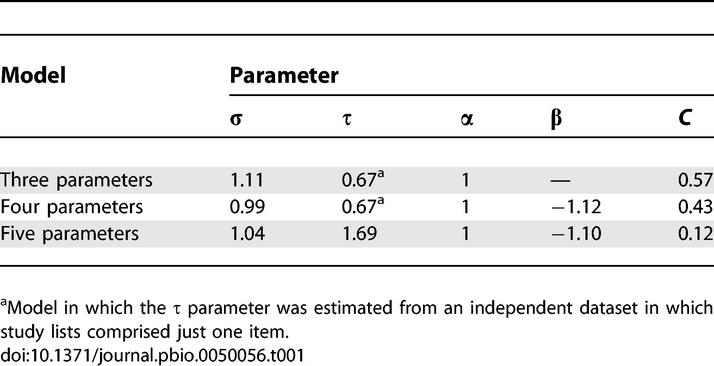
Parameter Values for Models with Three, Four, or Five Free Parameters

Interestingly, the *α* parameter was not significantly less than 1, indicating that in this experiment, when participants had to remember only two stimuli, both stimuli were remembered equally well. When participants must maintain more stimuli in memory, however, they are more likely to forget stimuli presented earlier in the list, as is shown in Figure 4.

Note that the *β* parameter remained negative and with a similar value regardless of model. Note that in both the four- and five-parameter models, *β* ∼ −1. This result is similar to that found by Kahana and Sekuler [1].

**Figure 8:**
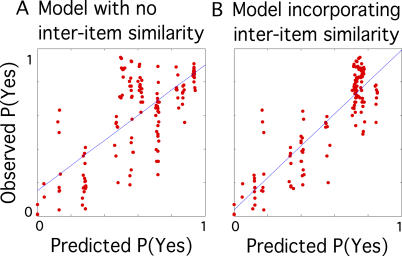
Model Fits for NEMo Model fits for NEMo are much better for versions including inter-item homogeneity (four-parameter model in [B]), than for identical models that do not include inter-item homogeneity (three-parameter model in [A]). Line indicates best fit to the data.

Results indicated that models must incorporate inter-item homogeneity in order to fit the data well. The three-parameter model that did not incorporate inter-item homogeneity (as shown in Figure 8A) accounted for only 51% of the variance (*r* ^2^), and had an Akaike information criterion (AIC) value (see Methods) of 1,010 (higher AIC values indicate worse fit [27]). On the other hand, the four-parameter model accounted for 78% of the variance (*r* ^2^), and had a considerably lower AIC value of 652. The five-parameter model, allowing *τ* to vary according to the list length 2 data, accounted for 81% of the variance (*r* ^2^) and had a slightly higher AIC value of 696, indicating that the addition of this extra parameter does not make the model more generalizable.

## Discussion

The ripple stimuli used here share many similarities with visual grating stimuli. Grating stimuli have long been a fixture of psychophysical experiments because they can be used to explore some properties of vision that are thought to be fundamental: spatial location, luminance, orientation, and spatial frequency. Similarly, the ripple sounds used in the present study can be used to examine some fundamental properties of hearing: frequency spectrum, sound level, and temporal frequency. The experiments presented here make use of the similarities to explore whether the fundamental information processing steps in vision and hearing are similar.

Moving ripple stimuli and visual gratings are processed by the nervous system in analogous ways, and therefore represent an important class of stimuli for comparing memory in the visual and auditory domains. Both auditory and visual cortical receptive fields have characteristic center-surround properties [7,9,15]. Further, edge detection in visual cortex appears to have an analog in auditory cortex [28]. Relatedly, both auditory and visual systems appear to exploit “sparse” coding [29]: when presented with stimuli of the appropriate type, individual cells respond very strongly to one example of the stimulus type and less strongly to other examples. In the visual modality, single primary visual cortical cells show large responses and specific tuning for oriented sine-wave gratings, or Gabor patches [9,30]. In the auditory modality, single primary auditory cortical cells show large responses and specific tuning for moving ripple stimuli [7,9,15].

Thus, early stages of cortical processing seem to treat Gabor patches and moving auditory ripples in an analogous fashion. Although a number of studies have examined recognition memory for Gabor patches [1,3], comparable tests of memory for auditory ripple stimuli have been lacking until now.

Parametrically manipulable stimuli were used in order to explore how memory alters the representation of stimuli. By using an auditory stimulus set for which early processing is similar to the visual gratings used here and in myriad previous studies (e.g., [1,20,30]), comparisons between memory effects in the two modalities can be made. Our results indicate that these auditory stimuli are processed in a way that is quite analogous to visual gratings. In Experiment 1, we directly tested properties of memory between the two modalities, and found little or no difference depending on stimulus type in how memory is affected by list length, retention interval, or serial position. The overall mean proportions correct were larger for the auditory stimuli than the visual stimuli, but the change with each of these variables was similar regardless of the stimulus type. In Experiment 2, we tested the hypothesis that a quantitative model for visual memory, NEMo, would fit the data for auditory memory better than other models. Indeed, NEMo fit the auditory memory data quite well, as shown in Figure 8, and the two major assumptions of the model proved true for auditory stimuli just as they had for visual stimuli: summed probe-item similarity and inter-item homogeneity each contribute to a participant's probability of responding that *Yes,* an item has been seen before.

### Direct Comparison between Memory for Auditory and Visual Stimuli

In our hands, direct comparison between auditory and visual memory revealed the two to be strikingly similar. The list length manipulation effectively changed the memory load participants had to bear, and has been used in experiments on vision [6] and hearing [11]. The current experiment reveals that the effect of load does not depend on the modality of the stimulus by comparing the stimulus types using the same participants and same experimental paradigm.

The effects of retention interval on recognition memory are also quite similar across stimulus types, as seen in Figure 3. Memory for auditory and visual stimuli decreased only modestly with retention interval. This result is consistent with previous studies of visual memory [20].

The effects of serial position on recognition memory were found to be quite similar across modality, as seen in Figure 4. Although this is consistent with some studies [31], there is an apparent contradiction in the literature: some researchers have found serial position curves of different shapes for auditory and visual experiments [32]. Many such experiments rely on auditory stimuli that are phonological in nature, and others use different experimental paradigms or stimulus types for auditory and visual experiments. A study by Ward and colleagues [31] implies that the auditory versus visual difference seen in other studies can be explained by the differing experimental methods used. When experimental methods are held constant, little or no serial position difference was seen between the two modalities, consistent with our data. Although there was no significant interaction of the effect of serial position with stimulus type, there is a trend toward a larger recency effect for the auditory stimuli than for the visual stimuli (Figure 4). The origin of this recency effect has been debated [33]. One idea put forward by Baddeley and Hitch [33] implies that the recency effect may be due to implicit learning of the items (similar to priming) followed by explicit retrieval of the residual memory.

Many studies using various types of stimuli in free-recall tasks have shown a “primacy effect” in which serial position 1 shows a better proportion correct than serial position 2 [34]. No such primacy effect is evident in our data, as can be seen in Figure 6. The lack of a prominent primacy effect is consistent with some previous experiments using this paradigm [1,35], whereas other experiments using the same paradigm, but different stimuli, have found modest primacy effects [36]. Previous experiments have shown that these effects are sensitive to the delay between the stimulus items and probe [14,37]. The absence of a primacy effect may be due to specifics of timing, the difficulty of rehearsing these stimuli, or an interaction of the stimuli and recognition memory task employed.

#### Differences between means.

Although the effects of list length, retention interval, and serial position were similar across the stimulus types, there is a striking and statistically significant difference between the mean proportion correct for the auditory and visual stimuli. Differences in mean in experiments like these may result from a difference in the overall difficulty of discriminating any two stimuli presented in the experiment. Although we performed a threshold test to determine each participant's just noticeable difference (JND) thresholds for each stimulus type, it is possible that these estimates erred on the side of being too easy for the auditory stimuli, despite the fact that JNDs were estimated using the same algorithm for all stimulus types.

Another possibility is that participants became gradually better at the auditory task, but not the visual tasks. Because the threshold tests were performed before the six experimental sessions, this would result in the auditory task becoming easier in later sessions, and a higher mean proportion correct. Analysis of participants' performance across session does not rule out this explanation. Text S1 explains the analysis that compares performance on trials early in the string of sessions to later trials. Participants' proportion correct increased with time in the auditory case, but not in the visual case, suggesting that participants improved on the auditory but not the visual task. Further experiments would be necessary to fully explore this differential learning effect. Although neither of these particular auditory and visual stimuli occur in participants' normal environments, it is possible that through prior exposure to stimuli like our visual stimuli, participants were better able to optimize their performance, but were less familiar with stimuli like the auditory stimuli.

### Summed Probe-Item Similarity

Figure 5 shows that summed probe-item similarity correlates very strongly with whether a probe will be judged as new. Because the similarity of the probe to the closest item is identical in each pair, the data imply that participants use information from all stimuli when making a judgment, not just information about the stimulus closest to the probe [1,25]. This gives credence to an exemplar model of memory, rather than a prototype model [25], and is entirely consistent with the results found in the visual domain [1,4,18].

### Inter-Item Homogeneity

These data indicate strongly that inter-item homogeneity plays a role in memory for sounds. When items in a list are more similar to each other, participants are less likely to say that a probe was a member of the list. This result was robust through direct data comparison (Figure 6) as well as by model fitting, which gave a more sensitive measure of the effect of inter-item homogeneity. As noted earlier, these results are consistent with experiments that examine memory for visual stimuli, including gratings and faces [1,4]. In fact, some older experiments using sound stimuli are consistent with this inter-item homogeneity effect. In one experiment, participants were required to remember a tone stimulus during presentation of distracter tones, and performed much worse when the distracter tones were presented both higher and lower than the remembered stimuli (low homogeneity between the remembered tone and the distracters), as opposed to the case when distracters were presented only higher or only lower than the remembered stimulus (higher overall homogeneity between the remembered tone and distracters) [38]. The similarity across stimulus type implies that the origin of the inter-item homogeneity effect is a process common to both auditory and visual memory.

### Similar Patterns Imply Similar Processing

The strikingly similar patterns of memory observed for the auditory and visual stimuli imply that the information-processing steps involved in memory for each stimulus type are similar. Previous research has shown that sensory-specific cortex is re-activated during memory for a sensation [39,40]. Further, lesions of some auditory-specific cortex results in impairment specifically to auditory memory [41]. The current data imply that the effects of inter-item homogeneity and summed probe-item similarity on memory either arise from non-sensory–specific cortex, or that the mechanisms in each sensory-specific region are very similar.

### Conclusion

The data presented here show that memory for visual and auditory stimuli obey many of the same principles. In both modalities, recognition performance changes in similar ways in response to variation in list length, retention interval, and serial position. Further, memory performance depends not only on the summed similarity between a probe and the remembered items, but also on the similarity of remembered items to one another. Memory performance data for both modalities are fit well by the NEMo. These results imply that auditory and visual short-term memory employ similar mechanisms.

Previous studies have examined how auditory and visual items are encoded into memory, implicating some structures in both visual and auditory working memory [42,43]. Behaviorally, visual and auditory stimuli can interfere with each other, indicating some shared processing [44]. On the other hand, some memory information is processed in sensory-specific cortex, indicating that the transformations performed on such information may differ between modalities [39,40,45]. Our data imply that, regardless of whether the processing is performed by the same brain area or not, similar processing is performed on auditory and visual stimuli as they are maintained and retrieved from memory.

For centuries, people have pondered possible parallels between their experiences of light and sound [17]. Belief that the two modalities were parallel probably influenced Sir Isaac Newton's conclusion that the visible spectrum contained seven colors, the same number of tone intervals in a musical octave [46]. (Newton observed: “And possibly colour may be distinguished into its principle degrees, red, orange, yellow, green, blue, indigo and deep violet, on the same ground that sound within an eighth is graduated into tones.” [46]) Today, 300 years after Newton, understanding of the neural signals supporting vision and hearing has advanced sufficiently that we have been able to formulate and test hypotheses about fundamental relationships between the characteristics of short-term memory for each modality.

## Materials and Methods

### Experiment 1.

Moving ripple sounds: moving ripple stimuli varied sinusoidally in both time (with a period *w* cycles per second [cps]) and frequency content (with a period *Ω* cycles per octave). The sounds were generated by superimposing sounds at many frequencies whose intensity at any time, and for any frequency *(f),* was defined by

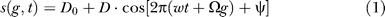
where *g* = log(*f/f*_0_), *t* is time, *ψ* is the phase of the ripple, and *D* is modulation depth. (*D*_0_ represents the baseline intensity, and is set to 1 in the equation to avoid negative intensity values.) *f*_0_ is the lowest allowed frequency. In these experiments, the parameter space was simplified by allowing only one parameter *(w)* to vary. Other parameters took the following fixed values: Ω = 1, *D*_0_ = 0.9, *f*_0_ = 200 Hz, and *ψ* was varied randomly between 0 and π/2 for each stimulus. Frequencies ranged over three octaves above *f*_0_ *,* that is, from 200 to 1,600 Hz. Choices for these parameters were made so that a range of stimuli with parameters close to these could be discriminated, as suggested by existing psychophysical data [10,47,48], and in pilot experiments of our own.

Each stimulus contained 20 logarithmically spaced frequencies per octave. Levels for each frequency were identical, but psychophysical loudness varied. However, the same group of frequencies was used for every stimulus, so the time-averaged loudness should be nearly identical for each of the stimuli. Equation 1 describes for each frequency *f,* a sinusoidal modulation of the level around some mean, at a rate of *w* cps. This produces a spectral profile that drifts in time, so that different frequencies are at their peaks at different times. Figure 1 illustrates the dynamic spectrum of a moving ripple, with modulation in both time (*w*, horizontal axis) and frequency content (Ω, vertical axis). For all stimuli, duration was fixed at 1 s. The level of the stimulus was ramped on and off gradually and linearly over 10 ms at the beginning and end of each stimulus. Frequencies at the spectral edges of the stimulus were treated identically to frequencies in the middle of the frequency range. Two examples of auditory stimuli with different *w* values are given in Audios S1 and S2, and correspond to the stimuli schematized in Figure 1A and 1B.

Visual stimuli: visual stimuli were Gabor patches, created and displayed using Matlab and extensions from the Psychtoolbox [49]. The CRT monitor was calibrated using Eye-One Match hardware and software from GretagMacbeth (http://www.gretagmacbeth.com/index.htm). The Gabor patches' mean luminance matched that of the background; the peak contrast of a Gabor patch was 0.2. Patches were windowed with a two-dimensional Gaussian envelope with a standard deviation of 1.4 degrees. Before windowing, the visual stimuli were generated according to the following equation:

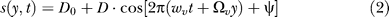
where *s* represents the luminance of the stimulus at any *y* (vertical) position and time, *t*. Note that these stimuli were aligned horizontally and moved only vertically; the luminance did not change with horizontal position. *ψ* is the phase of the grating, which varied randomly between 0 and π/2 for each stimulus. *D* is modulation depth. (*D*_0_ is the mean luminance, set to a mid-gray level on the monitor.) In these experiments, the parameter space was simplified by allowing only one parameter to vary at a time. In blocks with moving gratings, the *w*_v_ parameter varied; in blocks with static gratings, the spatial frequency, Ω_v_, parameter varied. Other parameters took the following fixed values: *D*_0_ = 0.9 and *f*_0_ = 200 Hz.

All moving gratings had a spatial frequency, Ω_v_ *,* of 0.72 cycles per degree, and moved with speeds that ranged upward from 1.5 cps (2.1 degrees per second). For static gratings, stimuli did not move (*w*_v_ = 0), and had spatial frequencies, Ω_v_ *,* with a minimum of 0.36 cycles per degree. An example of a moving grating is shown in Video S1, and an example of a static grating is shown in Figure 1C. Parameter values were chosen based on pilot experiments and previous data so that a range of stimuli with parameters near these would be discriminable.

Stimuli were tailored to each participant in an initial session, JND thresholds to achieve 70% correct were estimated using the QUEST algorithm [50] as implemented in the Psychtoolbox [49]. Participants were presented with two stimuli sequentially and responded indicating which stimulus was “faster” (in the case of moving ripples or moving gratings) or “thinner” (in the case of stationary gratings). Thresholds for each stimulus type were estimated in separate blocks. These JND values were used to create an array of ten stimuli for each participant, in which each stimulus differed from its nearest neighbor by one JND. All stimuli were chosen from this array, and were thus separated from one another by an integer number of JNDs.

The timing of stimulus presentation during threshold measurements was the same as that used in the later memory tests for a list with a single item. Stimuli were thus individually tailored for each participant, so that the task was of similar difficulty for all participants, and somewhat similar difficulty across modality [18]. The lowest value that each stimulus could take was the same for all participants. Other stimulus values were allowed to vary by participant in order to equate discriminability across participants. In Experiment 1, for the static grating stimulus type, in which the spatial frequency, Ω_v_ *,* changed, the lowest Ω_v_ value was 0.36 cycles per degree. For the moving grating stimulus type, in which temporal frequency, *w*_v_ *,* changed, the lowest *w*_v_ value was 0.025 cps. For the moving ripple sounds, the lowest possible ripple velocity, *w,* was 6 cps. In Experiment 2, the lowest ripple velocity, *w,* was 7 cps.

In order to minimize the possibility that participants could memorize all stimuli, a second, “jittered” set of stimuli was created and then used on half the trials chosen randomly. This list of stimuli started at 0.5 JND above the base value, and increased in units of 1 JND to create a second array of ten stimuli. For data analysis, we do not distinguish between trials on which the two arrays were used.

We experimentally manipulate the physical difference between any two stimuli, here measured in JND. However, the perceptual similarity is traditionally referred to in models that take perception into account. Therefore, when discussing physical stimuli, we refer to their difference (in JND), but later, when discussing fits to models, it is the related perceptual similarity that is relevant.

Participants: participants for all experiments were between the ages of 18 to 30 y, and were recruited from the Brandeis student population. They participated for payment of $8 per session plus a performance-based bonus. Using a MAICO MA39 audiometer, participants' hearing thresholds were measured at 250, 500, 750, 1,000, 2,000, 3,000, 4,000, and 6,000 Hz. Each participant had normal or better hearing, that is, thresholds under 20 dB_HL_ (decibels hearing level) at each frequency.

Fourteen participants participated in seven total sessions each. In an initial session, hearing was tested and vision was tested to be 20/20 or better (using a Snellen eye chart), participants performed 30 practice trials for each stimulus type, and JND thresholds were measured at a 70% accuracy level for each stimulus type. Each of the subsequent six sessions lasted approximately 1 h, and consisted of 504 trials. A session began with 15 practice trials, whose results were not included in subsequent data analysis. For each participant, successive sessions were separated by at least 3 h, and all sessions were completed within 2.5 wk.

Apparatus and sound levels: participants listened to ripple sounds through Sennheiser Pro HD 280 headphones. All stimuli were produced by Apple Macintosh iMac computers and Matlab, using extensions from the Psychtoolbox [49]. Sound levels for this system were measured using a Knowles electronic mannequin for acoustic research, in order to define the stimulus intensity at the participant's eardrum. Levels for all stimuli in Experiment 2 were 79 dB_SPL_ (decibels sound pressure level), well above our participants' hearing thresholds, and levels for stimuli in Experiment 1 were similar (with the same code and hardware settings, but a different computer).

This experiment examined and compared some basic characteristics of short-term memory for moving ripple sounds and for Gabor patches. Using Sternberg's recognition memory paradigm, we examined recognition's dependence on the number of items to be remembered, the interval over which the items had to be retained, and the serial position of the to-be-remembered item [6]. The experiment used static visual gratings (in which the spatial frequency of the gratings, Ω_v_ *,* varied), moving visual gratings (in which the speed of the gratings, *w*_v_ *,* varied), and moving ripple sounds (in which the temporal frequency, *w,* of the ripples varied).

Stimulus presentation: trials were presented in blocks such that only one stimulus type (moving ripple sounds, static gratings, or moving visual gratings) was presented per block. During presentation of either visual or auditory list stimuli, participants fixated on a “+” in the center of a computer screen. Each stimulus, auditory or visual, lasted for 1 s. After the last item from a list was presented, a short beep sounded, and the “+” was replaced by the text “...”, indicating that the participant should wait for the probe. The text “?” was presented onscreen during presentation of the probe (for sound stimuli only) and after the probe presentation, before the participant made a response. Participants were instructed to be as quick and accurate with responses as possible. Stimuli were presented in blocks of 84 trials of a given stimulus type. Six total blocks were presented per session. The first two trials of each block were not used for analysis to allow for task-switching effects.

Stimuli for each list were chosen from a set created as described above for each participant based on their own JND threshold. Trials with different list lengths and retention intervals were randomly interleaved. Twenty-four trials of each possible serial position were presented to each participant, for each stimulus type. Effect of retention interval was examined by having participants perform trials in which a single stimulus was followed by a probe, after a retention interval of 0.6, 1.9, 3.2, 4.5, or 9.7 s; 24 trials of each retention interval were performed by each participant. Equal numbers of trials in which the probe matched a list item (target), and trials in which the probe did not match (lure) were performed.

Trials were self-paced, with each beginning only when participants indicated with a key press that they were ready. Participants were alerted with a high or low tone whether they got the current trial correct or incorrect, and were updated after each trial as to their percent correct. For every percentage point above 70%, participants received an extra $0.25 reward above their base payment of $56.

### Experiment 2.

Participants and stimulus presentation: on each trial, a list of one or two ripple stimuli (*s*_1_, *s*_2_) were presented, followed by a probe (*p*). As in Experiment 1, the participants' task was to identify whether the probe stimulus matched any of the items presented in the list, and press a button to indicate a choice. During list presentation, participants fixated on a “+” in the center of a computer screen. This was replaced by a “?” during the presentation of the probe item. Twelve participants participated in each of eight sessions, following an initial session in which hearing was tested, JND thresholds for the *w* parameter (cps) were measured, and 200 practice trials were performed. Sessions were approximately 1 h each, and consisted of 586 trials. At the beginning of every session, each participant completed at least 30 practice trials that were excluded from data analysis. Each session began at least 6 h from the previous session, and all sessions were completed within 3 wk. All other details are as described for auditory stimuli in Experiment 1.

Summed probe-item similarity: in order to examine the effect of summed probe-item similarity independently of other confounds, such as the similarity of the probe to the closest item or the inter-item homogeneity, stimulus conditions were created that varied summed probe-item similarity while other factors were held constant. Two pairs of conditions were created that were similar in all respects, but the summed probe-item similarity varied between the two conditions in the pair.

Figure 5A shows the relationships between stimuli for each condition. All figures indicate relationships between stimuli in terms of their differences in units of JND.

Pairs of conditions (labeled a & b on one side, and c & d on the other) were created with identical inter-item homogeneities, and identical similarities between the probe and the item closest to it. However, each pair has one low and one high summed probe-item similarity (pair a & b, for example, both have inter-item difference = 2 JND, but summed probe-item differences of 2 and 4 JND units, respectively).

Figures 5 and 6 indicate only the relationship among the stimuli in units of JND, not their physical values. Part A in these figures illustrates the case when *s*_1_ < *s*_2_, equally often *s*_1_ > *s*_2_. Also in conditions b and d, the probe, *p,* is equally likely to be greater than or less than the stimuli *s*_1_ and *s*_2_. The conditions as shown in the figures do not specify exactly the stimulus values for a trial. Eight cases of each condition were chosen randomly from all possible configurations that satisfy the condition, given ten stimuli in the array. This made 64 lure cases. Twenty repetitions of each case were performed by each participant, interleaved among the other trial types. For each lure case, analogous target cases were created where the probe matched one of the stimuli. Each target case matched a different lure case in either inter-item homogeneity (in conditions a–d), or summed probe-item similarity (in conditions e–h, explained below).

Inter-item homogeneity: stimulus conditions with high and low inter-item homogeneity were created according to Figure 6A, which follows the same conventions as Figure 5A. Relationships between stimuli for each condition are shown in terms of their physical differences, in units of JND. Two sets of paired high and low homogeneity conditions were created; both members of a pair had the same inter-item homogeneity and similarity between the stimulus and the closest probe.

Computational modeling of results: fitting computational models to experimental data can help determine what information processing steps are involved in short-term memory. Previous experiments in the visual domain found that a NEMo, including effects of summed probe-item similarity as well as inter-item homogeneity, fit data for short-term visual memory well [1,2,4].

The NEMo model was applied only to the data from the 128 auditory memory cases whose list length was two items, because only those trials incorporated information about inter-item homogeneity, important to the model. The NEMo assumes that given a list of *L* items and a probe item, *p,* the participant will respond that “*Yes,* the probe is a member of the list” if the quantity:

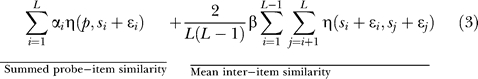
exceeds a threshold criterion value, *C*.

The first term depends on the summed similarity between the probe and the items on the list. *α* is defined as 1 for the most recent stimulus; its value for a less-recent stimulus determines the degree of forgetting of that stimulus. It should take on values less than 1 if the earlier item is forgotten more readily. *η,* as defined in Equation 4, measures the perceptual similarity between any two stimuli, as a function of *τ,* which defines how quickly perceptual similarity drops with physical distance:

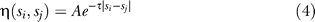



The parameter *A* in Equation 4 defines the maximum similarity between two stimuli. *ɛ* defines the noise in the memory representation of the stimulus (hence the label “Noisy Exemplar”). The parameter *ɛ* is a normally distributed random variable with variance *σ* ^2^. Note that the similarities incorporated in the model depend on the noisy values of the remembered stimuli.

The second term in Equation 3 involves the homogeneity of the list, that is, the similarity between the remembered list items. *β* is a parameter determining the direction and amplitude of the effect of list homogeneity. If *β* < 0, as was found in earlier experiments using visual stimuli, a given lure will be more tempting when *s*_1_ and *s*_2_ are widely separated; conversely, if *β* > 0, a lure will be less tempting when *s*_1_ and *s*_2_ are widely separated. If *β* = 0, the model does not depend on inter-item homogeneity, and is a close variant of Nosofsky's Generalized Context Model [24]. The parameter *A,* as defined in Equation 4, was set to 1. This model allows five parameters, *σ, α, β, C,* and *τ,* to vary.

Two additional similar models were also examined. A second model assumes that the similarity between items can be predicted from participants' probability of confusing two items in a trial of list length 1. This model adopts values for *τ* and *A* for each participant based on the fit of Equation 4 to their data with list length 1. This model is identical to that above, but simpler, allowing only four parameters (*σ, α, β,* and *C*) to vary based on the data with list length 2.

A third model is identical to the second, but in it, *β* is forced to be 0, which means that only three parameters are free to vary: *σ, α,* and *C*. Note that this last model does not take into account any possible influence of inter-item homogeneity. Models are labeled according to the number of parameters varied in each: five, four, and three.

Model fits: models were fit to participants' accuracy data by means of a genetic algorithm. Such a method was chosen because it is robust to the presence of local minima [51]. The parameter spaces involved in this experiment are relatively complex, so the genetic algorithm approach was particularly attractive. To summarize our implementation of a genetic algorithm, 3,000 “individuals” were generated, each a vector of randomly chosen values for each of model's parameters. The ranges for each parameter were: 0 < *σ* < 5, −3 < *τ* < 3, 0 < *α* <1, −2 < *β* < 2, and 0 < *C* < 2. Three thousand trials were simulated for each individual, each with a randomly chosen value for *ɛ* given the parameter *σ*. When the value in Expression 3 exceeds *C,* the simulation produced a *Yes* response. The proportion of *Yes* responses for each case was calculated. The fitness of each individual was computed by calculating the log likelihood that the predicted and observed data came from the same distribution. Log likelihood was chosen because it is more robust to non-normal data than is a least-squares error method [27]. The 10% most fit individuals are maintained to the next generation. These act as “parents” to the next generation: the parameters for the 3,000 individuals of the next generation come from combinations of pairs of parents and mutations. This procedure was repeated for 25 generations. Best-fit parameters typically did not change past the 20th generation, indicating stable parameter values had been obtained.

Model comparison: in order to compare the three models described above, the predicted data and observed data were plotted against each other, and a measurement of the variance accounted for by the model, *r* ^2^ *,* was calculated. However, when comparing two models with different complexities, for example, with different numbers of parameters, the important distinction between models is their generalizability to new data, that is, the likelihood that the model will fit another set of similar data. The AIC is a measure of model fitness that takes into account both how well the data fit the model and the number of parameters in the model. See the work of Myung et al. [27] for more information about AIC and calculation techniques. Thus, both the AIC and *r* ^2^ values were used to discriminate between different models.

## Supporting Information

Audio S1 Auditory Ripple Sample (Faster) An example of one auditory ripple sound used. This sound is “faster” than the other auditory ripple example (Audio S2), and corresponds to Figure 1A with *w* = 16 Hz.
(31 KB WAV)

Audio S2 Auditory Ripple Sample (Slower) An example of one auditory ripple sound used. This sound is “slower” than the other auditory ripple example (Audio S1), and corresponds to Figure 1B with *w* = 8 Hz.
(31 KB WAV)

Text S1 Possible Differential Learning Effects for Auditory and Visual Data (95 KB PDF)

Video S1 Moving Visual Grating Demo An example of the moving visual grating stimulus.
(2.7 MB MOV)

## Abbreviations

AIC: Akaike information criterion
ANOVA: analysis of variance
cps: cycles per second
JND: just noticeable difference
NEMo: Noisy Exemplar Model
