# Supplementary material for: Auditory Short-Term Memory Behaves Like Visual Short-Term Memory
Source: PLoS Biol. 2007 Feb 20;5(3):e56. doi: 10.1371/journal.pbio.0050056 (PMC1800308; doi:10.1371/journal.pbio.0050056)
Supplement: Supplementary file 1 [file pbio.0050056.sd001.pdf]

Auditory Short-Term Memory Behaves Like Visual Short-Term Memory

September 18, 2006

Kristina M. Visscher,† Elina Kaplan,† Michael J. Kahana‡ and Robert Sekuler†

†Volen Center for Complex Systems, Brandeis University,
‡Department of Psychology, University of Pennsylvania,
¶ Corresponding author information: Brandeis University, Volen Center for Complex Systems,
415 South Street, MS 013
Waltham, MA 02454-9110
Email:visscher@brandeis.edu, Phone:781-736-3266

Supplementary online material

Possible differential learning effects for auditory and visual data

Subjects had greater average proportion correct for the auditory task than for either visual task. This could be because they are somehow better at the auditory task, worse at the task used to determine auditory threshold, or because they got better at auditory discrimination as the task wore on. Because the task used to determine auditory threshold was almost identical to the auditory task, the second possibility is unlikely. In the third possibility, subjects would have been poorer at auditory discrimination when their threshold was estimated than later on in the task, meaning that their effective JND would be artificially too lax for these subjects. To investigate this, we looked at how proportion correct on each task changed over time.

Data were divided into 12 time bins (the beginning and end of each of the 6 sessions). Only list length 1 cases were included in this analysis for simplicity and to take out any effects of serial position or list length. Data are shown in Figure 9 in Supplementary Online Materials. Subjects' proportion correct increased with time in the auditory case (last quarter of data has 8% greater proportion correct than first quarter) and not in the visual cases (3% less and 0.5% greater for grating speed and width judgments respectively). This difference is confirmed by repeated measures ANOVA (3 stimulus types by 12 time bins repeated measures ANOVA reveals an interaction between the two with $F(33,429)=1.826$, $p=.015$), suggesting that subjects got better at the auditory task, but not the visual tasks.

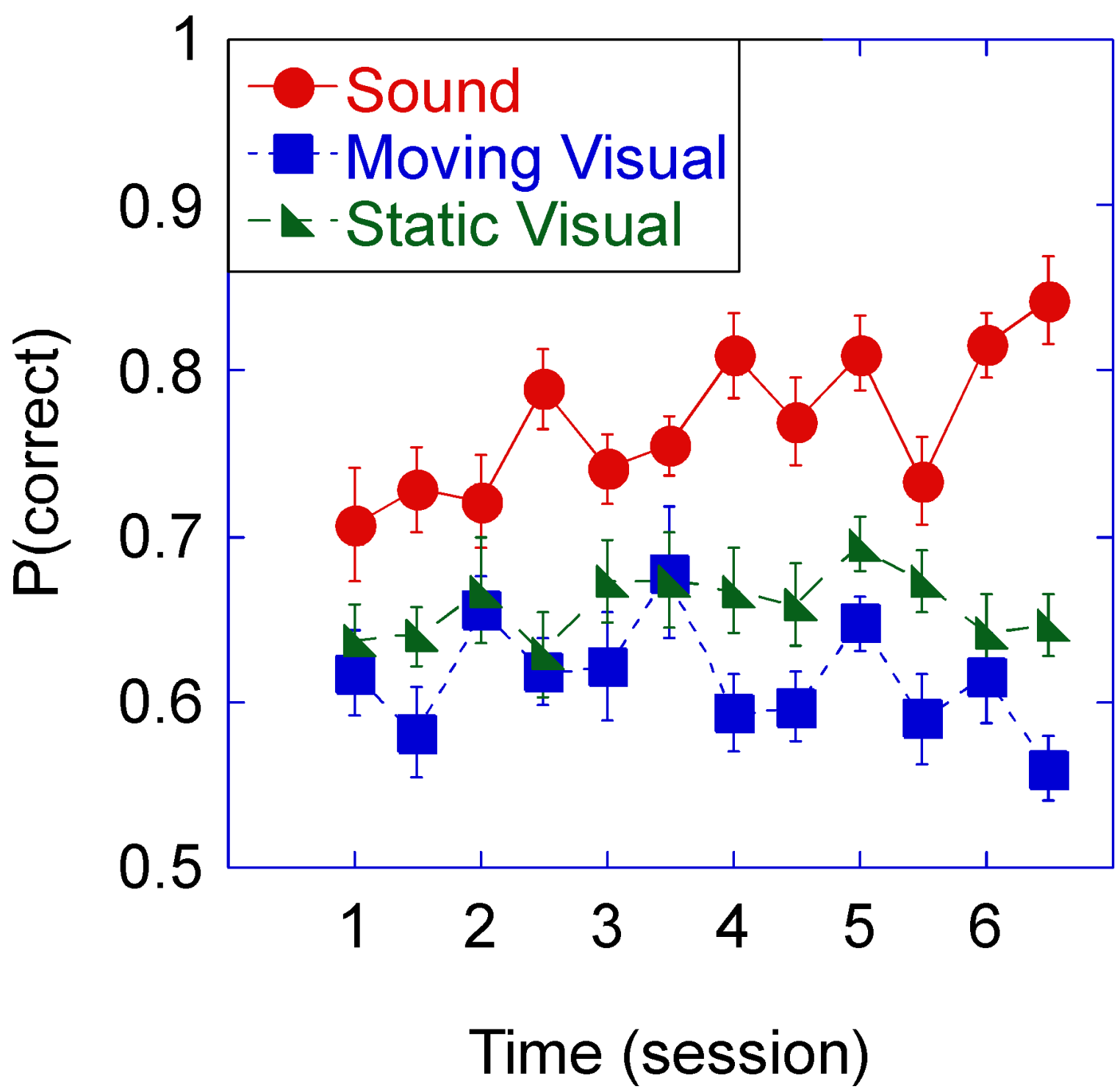

Figure 1: Effect of practice on proportion correct for Experiment 2. Trials are binned so that each data point represents the first or last half of a single session. Error bars are within subject standard error of the mean.

As a somewhat related aside, two subjects who had participated in previous experiments using ripple sounds showed no such 'learning effect' (mean performance for auditory stimuli got 3% worse, while visual stimuli got 1% and 4% better respectively, there was no significant interaction among stimulus types $F(33,33)=0.8$, $p=0.65$), indicating that they had already optimized their performance for the auditory stimuli at the time of the threshold test.

Further experiments would be needed to determine whether the differential learning effect arises from subjects' overall greater familiarity with stimuli like the Gabor patches, or from some intrinsic difference between memory for the two stimulus types. In the current experiment, we cannot differentiate the possibility that the auditory task and stimuli are inherently learned more slowly from the possibility that the auditory task used stimuli with which these subjects had less prior experience.
